# The Influence of Mechanical Stress Micro Fields around Pores on the Strength of Elongated Etched Membrane

**DOI:** 10.3390/membranes12111168

**Published:** 2022-11-21

**Authors:** Venera Gumirova, Irina Razumovskaya, Pavel Apel, Sergey Bedin, Andrey Naumov

**Affiliations:** 1 Laboratory of Advanced Materials Physics, Moscow Pedagogical State University, 119991 Moscow, Russia; 2 Flerov Laboratory of Nuclear Reactions, Joint Institute for Nuclear Research, 141980 Dubna, Russia; 3 The Lebedev Physical Institute of the Russian Academy of Sciences, Troitsk Branch, Troitsk, 108840 Moscow, Russia; 4 Laboratory for Spectroscopy of Electronic Spectra of Molecules, Institute for Spectroscopy RAS, Troitsk, 108840 Moscow, Russia

**Keywords:** track-etched membranes, pores, mechanical properties, microcracks, polymers, stress concentration, distribution functions, fractal dimension, wavelet analysis

## Abstract

The investigation of the mechanical properties of polymer track-etched membranes (TMs) has attracted significant attention in connection with the extended region of their possible applications. In the present work, the mechanical stress fields around the pores of an elongated polyethylene terephthalate TM and around the 0.3 mm holes in model polymer specimens were studied in polarized light and with the finite element method. A break-up experiment showed the controlling role of stress field interaction in the forming of a microcrack system and the generation of a main crack with rupture of the TM (or model pattern). This interaction depended on the relative distance between the pores (holes) and their orientation. The results of the calculations of the pore distribution function over the surface of the TM via the net method and wavelet analysis are presented. The fractal character of pore distribution was established. The geometric characteristics of the TM pore system as initial defects are inherited by obtaining TM-based composites.

## 1. Introduction

Polymer membranes are widely used in many separation processes such as microfiltration, ultrafiltration, reverse osmosis, nanofiltration, and electrodialysis. Very often, a membrane is exploited under hydraulic pressure, which may cause deformation and even violate the integrity of the membrane. For this reason, questions regarding the membranes’ mechanical properties and the mechanisms of the membranes’ deformation and mechanical failure are of primary importance for practical use. These aspects can be studied both experimentally and theoretically [1,2,3,4,5,6,7,8,9,10].

Regarding the experimental approach, membranes with a regular pore structure, such as track-etched membranes (TMs), seem to be the most promising model. Track-etched membranes, which are produced by irradiating a polymer foil with heavy ions and chemical etching, possess arrays of uniform pore channels and can be regarded as a good model for studies of this kind [11,12,13,14].

Recently, their scope of application has been radically expanded, as they have proved to be a promising matrix for the creation of polymer/metal nanocomposites with unique properties, as well as a template for the synthesis of nanowires, nanotubes, nanocables, and others [15,16,17,18,19]. Depending on application, track membranes can be fabricated from different materials such as polyethylene terephthalate, polycarbonate, polyimide, polypropylene, and polyethylene naphthalate [11]. Each material imparts a specific combination of chemical, mechanical, and physical (electrical, optical, and structural) properties to the membrane, which makes it possible to obtain a wide variety of pore structures and realize completely different functions. Their main functional property (filters remains) enriched with additional capabilities, which open the pay to such applications as, for example, diffraction X-ray filters [19], mass spectrometry [20], structures for adsorber and ultrafiltration [21,22], metasurfaces for SERS analysis [23,24,25,26,27]. Polyimide TMs decorated with polyvinylpyrrolidone were used as separators for a self-assembling protective layer on a lithium-metal anode [28]. Track-etched membranes obtained via the irradiation of polymer films with heavy ions and the subsequent etching of latent tracks can be applied in many fields, including biomedicine. In [29], the resistive-pulse sensing of DNA and the characterization of DNA translocation were performed by a polymeric nanopore sensor. New methods of TM surface analysis are appearing, e.g., in [30], estimations of porosity, pore localization, and pore number were carried out with the use of machine learning.

The mechanical properties of usual TMs are not under special consideration. They were partly studied in [7], where TMs were used in implantable medical devices. Many investigators have studied and simulated the process of TM single-axis extension for filtration characteristic improvement, though without an emphasis on strength [3,4,8]. In [10], the influence of radiation on strength of polymer films comprising PET and PEN was investigated. The pore’s role in fractures in the all these works was insufficiently considered.

In this regard, questions remain regarding TM strength and its effect on the fracture of TM pore systems—the starting concentrators of mechanical stress. The greater the porosity, the higher the permeability of a TM. However, increasing porosity leads to a reduced strength and possible fractures during operation.

The pores of a TM affect its strength due to the following factors:

(1) The reduction in the working section of a sample in a real single-axis, elongated cross-section, which is smaller than that of a normal cross-section because of pores.
(2) The concentration of mechanical stresses on the pores, as on each defect of a solid structure, the local mechanical stress is more than a normal structure’s stress.
(3) The interaction of elastic stress fields around closely spaced pores.

Usually, only the first factor is taken into account. However, experimental [5,6] data indicate the importance of the other two.

## 2. Materials and Methods

In the experimental part of this work, the deformation properties and strength of a TM created at the G.N. Flerov Nuclear Reaction Laboratory (Dubna, Russia) (obtained from a biaxially oriented polyethylene terephthalate (PET) film with a thickness of 12 μm and irradiated by high-energy *Kr* ions at 1–2 MeV/nucleon) were investigated. The pores formed a system of parallel disjoint channels perpendicular to the surface of the TM. With a constant surface density of pores *n* = 4 × 10^6^ cm^−2^, their diameter *d* was varied. The variation of the diameter was achieved with different track-etching times (Table 1). Reductions in film thickness over the etching time were taken into account in the strength tests.

When discussing the results of experiments and statistical calculations, the authors refer to the individual results obtained for TMs with other characteristics (Table 2).

The pores were measured to provide an average diameter value.

The strength of the TM was determined in a single-axis tensioning regime on an Autograph AGS-5kN universal tensile testing machine from Shimadzuat (Kyoto, Japan) a tensile speed of 2 mm/min. The samples used to measure the strength were strips with a working part size of 5 mm × 30 mm cut off with use of a REY RAN manual cutting press. Each point on the strength plot corresponded to at least 15 samples.

In the natural simulation of the stress fields around the pores, samples of 0.04 mm thick polyimide film with artificially applied round holes (0.3 mm diameter) were used. The holes were applied using a Proxxon FBS 240/E (Niersbach, Germany) drilling unit consisting of an FBS 240/E drilling machine with a rotation speed of 5000 to 20,000 rpm, a Proxxon MB 140S drill pedestal with a rigid return spring, a Proxxon CT 70 coordinate table, and a pair of step clips. The thickness of the samples was measured with a PosiTector (DeFelsko Inspection Instruments, Ogdensburg, NY, USA) 6000 magnetic thickness gauge corrected to 1 μm. The elastic stress fields around the holes were observed with an Altami-Polar (Al’tami, St. Petersburg, Russia) 312 polarizing microscope. The TM was also photographed with a Nikon LV100 (Tokyo, Japan) optical microscope and Tesla BS340 (Prague, Czech Republic) scanning electron microscope (SEM).

Optical and SEM photographs of the membrane surfaces were used as material for computer analysis. Each surface image was described as a matrix, where the column and row numbers replace coordinates and the cell value is proportional to the amplitude of the surface signal.

When determining the pore distribution function over the surface of the TM, a hypothesis was formulated regarding its conformity with a Poisson distribution or a normal distribution. The classical function of the pore distribution at the smallest distances to the nearest neighbors was considered. Since the clusters of high pore densities lead to rapid TM rupture, the functions of distribution at the smallest distances up to four and six nearest neighbors were also considered.

The fractal dimension (FD) was calculated using the wavelet analysis method and the net-point method. The calculation programs were written in MATLAB 6.0 and debugged on fractal structures with known dimensions.

The COMSOL Multiphysics 6.0 packet and the net-point method were used to simulate the interaction of mechanical stress fields around the closely spaced TM pores in the case of a plastic–elastic problem.

The phase field method (one of the methods for simulating crack propagation in COMSOL Multiphysics 6.0) was used to obtain the most probable crack trajectories under a single-axis TM tension.

## 3. Results and Discussion

The first influence factor of pores (reduction in the working section of the TM) can be considered [5] using a formula similar to the well-known Smith–Nielsen formula for composites. A TM can be considered a composite with zero adhesion between its matrix and filler, and its strength or yield limit (only considering the working section reduction) is equal to

(1) $$\sigma =\sigma_{m}\left(1-\sqrt{P}\right)$$

where *σ* and *σ_m_* are the strength or yield point of the TM and matrix, respectively, and *P* is the TM porosity.

The 2D approximation of our problem caused the porosity in Formula (1) to be in the 1/2 power (instead of the 2/3 power in the Smith–Nielsen formula). At small P variation limits, the experimental strength dependence *σ*(*P*) may appear to be linear [2].

In addition, we previously introduced [5] the effective stress concentration factor β into the formula, thus considering the second pore influence factor:

(2) $$\sigma =\frac{\sigma_{m}}{\beta }\left(1-\sqrt{P}\right)$$

The effective stress concentration coefficient could be determined from the experiment by comparing the strength of the TM and the original film (Table 3, Sample 6) and by considering the reduction in the working section by both pores and etching.

**Figure 1:**
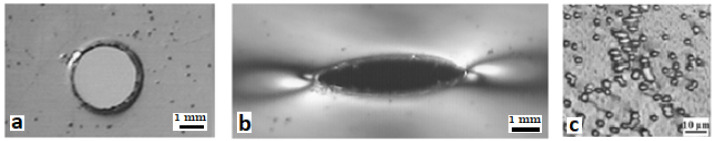
(**a**) Modeling of the pore with a 0.3 mm hole drilled into the polyimide film; (**b**) distortion of the hole shape at a tensile rate of 2 mm/min to 100% deformation; (**c**) elongation of the pore shape of the TM with *d* = 4 μm and *n* = 10^6^ cm^−2^ at a 10% deformation.

The value of β was 1.4–1.6. The same value was obtained for a few other TMs [5]. Additionally, it is known that for cylindrical pores perpendicular to the surface of a TM, the coefficient becomes β = 3. The reason for the lower value of β is the evolution of the pore shape when the TM is elongated. This was confirmed by model experiments with artificial holes in the polyimide film (Figure 1a,b) and microphotographs of the elongated TM (Figure 1c).

A comparison of the photographs in Figure 1a,b allows us to see the hole evolution in the process of single-axis extension.

**Figure 2:**
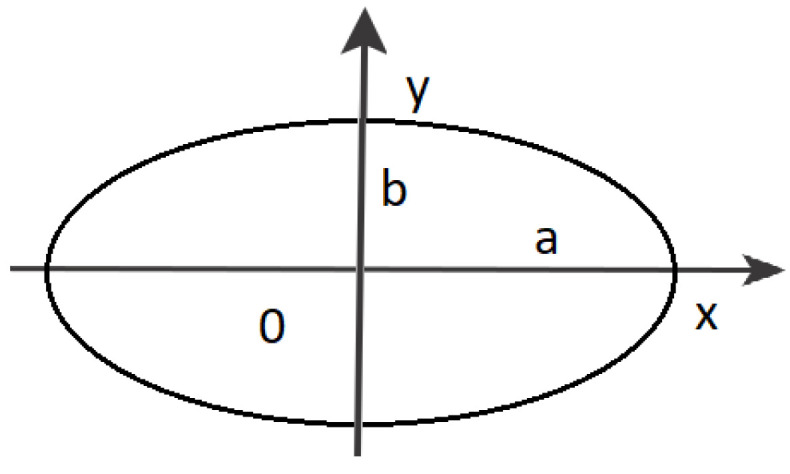
The model of the form changes for circular pores in elongated TM.

If we approximate the elongated pore by an ellipse (Figure 2) and use the known formula

(3) $$\beta =1+2\sqrt{\frac{l}{\rho }}$$

where *l* is the length of the crack and *ρ* is the radius of curvature in the vertex, then the coefficient of stress concentration for the elongated pore is equal to

(4) $$\beta =1+2\sqrt{\frac{b^{2}}{a^{2}}}=1+2\frac{b}{a}$$

where *a* and *b* are the axes of the ellipse. TM deformation leads to a reduction in the length of the crack-pore (the small half-axis of the ellipse) and an increase in the effective radius of its curvature, i.e., a reduction in *β*.

So, the concentration of stress on the pores and its evolution are significant factors in the deformation and fracture of TMs.

**Figure 3:**
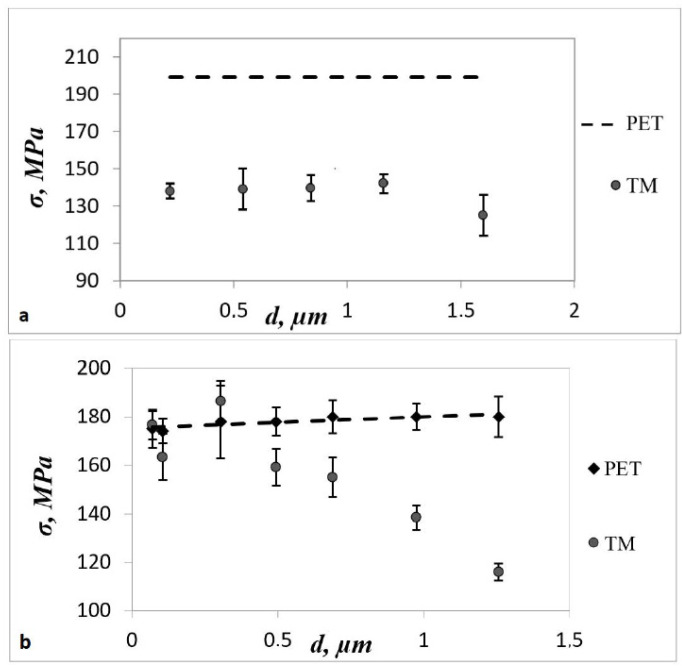

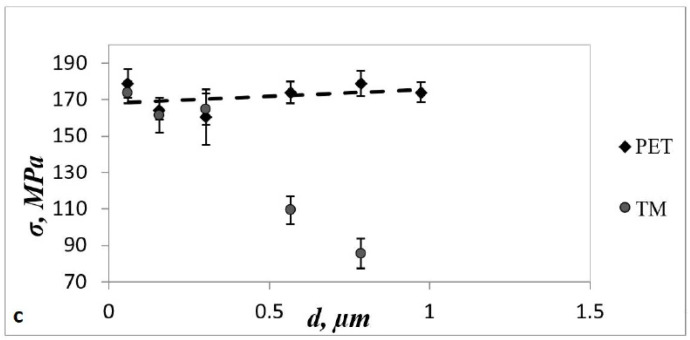
Dependence of strength on pore diameter for TMs with different densities: (**a**) *n* = 4 × 10^6^ cm^−2^; (**b**) *n* = 1.18 × 10^7^ cm^−2^; and (**c**) *n* = 4.41 × 10^7^ cm^−2^. The dotted line on all plots corresponds to the unirradiated PET sample (without pores). For (**b**,**c**), it was considered that the film had passed the same etching mode as the TM with this diameter value.

At the same time, an increase in the *β* coefficient and a decrease in the strength and the yield point were detected with an increase in the pore diameter. We observed this effect for other TM pore densities (Figure 3). The reason seems to be the third factor of influence of pores on the deformation-strength properties of TMs—the complex interaction of elastic stress fields around closely located pores.

The third factor, the interaction of elastic stress fields around closely spaced pores, presents the greatest difficulties when analyzing the role of pores in the fracture of TMs. However, just it determines the development of the mass of microcracks in TMs, culminating in the growth of main cracks.

The interaction of elastic stress fields around pores begins when the distance between them is less than 5 of their diameters. The simplest criterion for the presence of the interaction of pores for TMs with pore diameter d and surface density *n* is the condition for the average distance R between pores equal to *n*^1/2^:

(5) $$R=n^{1/2}<5d$$

Table 4 shows that for $\overset{\text{¯}}{r}$, corresponding to regular network, samples 4 and 5 satisfied condition (5). Sample 5 had a lower strength and yield point than the other samples, but this did not apply to sample 4 (Figure 3). However, as is shown below, the mean value of the pore distances determined by the formula *R* = *n*^1/2^ was only an approximation, strictly valid only for a regular pore network. Table 4 shows the relative average distances calculated using the different methods described below.

The difference in the value of the mean distances for columns 3 and 5–7 demonstrates the applicability of a regular network model to the pores of the presented TM for the calculation of this parameter.

**Figure 4:**
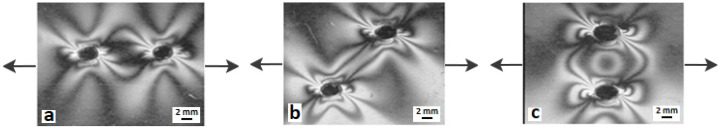
Fields of elastic stresses around holes in polyimide film with diameter *d* = 0.3 mm applied at different angles to the direction of tension; from left to right: (**a**) 90°, (**b**) 45°, and (**c**) 0°. Tensile stress was 15 MPa. The direction of the tensile stress is indicated by needles.

The interaction of the pores was simulated by the interaction of the circular holes that were artificially applied to the polyimide film at three different positions in relation to each other and to the direction of tension (Figure 4). At the sample width of 10 mm, the distance of the holes to its edges exceeded 13 diameters. The distance between the centers of the holes in all cases was 15 mm, that is, equal to 5 of their diameters. In polarized light, the shear fields around the holes were observed at different stresses (Figure 4).

The behavior of stress fields in the neighborhood of circular holes is an important subject of study in the theory of fracture. The calculation and analysis of the strained state of a plane with two or more circular holes were particularly presented in [31]. Our results qualitatively correspond to the conclusions of that work.

With an increase in the stress applied to the film, the overstress area of the holes and the probability of their interaction increased. For the holes located at an angle of 45°, the field interaction occurred at a lower stress.

Accordingly, the strength of the samples with the holes proved to be dependent on their orientation relative to each other. The samples with two holes at an angle of 45° to the direction of tension broke at an average stress of 112 MPa, the samples with holes at an angle of 90° to the direction of tension broke at an average stress 118 MPa, and the samples with holes at an angle of 0° broke at an average stress of 128 MPa. The sample with no holes had a strength of 190 MPa.

The strength of the sample with a single pore was determined by the first and second factors (reduction in the working section due to the holes and stress concentration). It was equal 161 MPa, which corresponded to *β* = 1.2 (Table 5).

In reality, to estimate the role of the interaction of a great number of TM pores, it is necessary to know the function of their surface distribution. Note that knowledge of the pore distribution over a TM surface is also essential for the use of structures produced on its base. For example, the pore distribution determines the overlap of electrical fields of replica emitters and the formation of hot spots for SERS spectroscopy on an array of metal nanowires grown on TM. Pore distribution also determines the metal filler distribution on micro- and nanocomposite matrix synthesis based on TM.

Here, the pore distribution and interactions were analyzed with computer calculations and experiments. Since pore channels are perpendicular to the surface of a TM, a two-dimensional problem could be considered.

The pores were non-uniformly distributed over the surface, and a visual analysis of TM photomicrography showed that there were clear areas with a low pore density (in the form of winding tracks) and clusters of closely spaced pores (in the form of chains). The nature of these structures is not discussed in this article, but an examination of the studied TM’s distribution in relation to that of a random one is warranted.

The classical method of testing the hypothesis for agreement with the Poisson (random) distribution of a discrete quantity, in this case the pores over the surface of the TM, is Pearson’s (chi-squared) *χ*^2^ test. The studied area of the surface of the TM was divided into squares of equal area, and the frequencies of several pores (centers of pores) in each square were calculated. The values of the number of pores regulated in ascending order formed a sample. The criterion for the selection of the area of one square was that the number of frequencies below 5 was not more than 20% of the total number of frequencies. The preliminary number of squares on which the surface of the TM was partitioned was determined by the Sturges formula: *m* = 1 + 3322·*lg* (*N*), where *N* is the number of pores on the surface [9]. A Poisson distribution is characterized by the equality of its mathematical expectation *a* and variance *D*, and the probability that a random quantity *X* takes the value *k* is expressed by the formula:

(6) $$p\left(X=k\right)=\frac{a^{k}}{k!}e^{-a}$$

The obtained value of the Pearson criterion *χ*^2^ was compared with the table value $\;\chi_{table}^{2}$ at a given level of significance. If $\;\chi^{2}<\chi_{table}^{2}$, then there was no reason to reject the hypothesis regarding Poisson law for the pore distribution over the surface of the TM.

**Figure 5:**
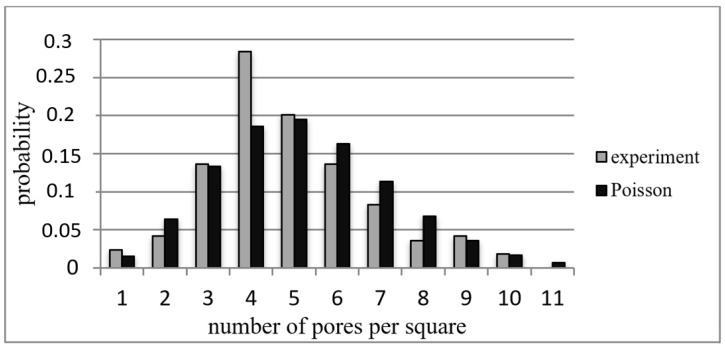
Histograms of pore distribution over TM surface; *n* = 10^6^ cm^−2^ and *d* = 4 μm.

As an example, Figure 5 shows histograms for experimental and theoretical pore distributions for the TM with *n* = 10^6^ cm^−2^ and *d* = 4 μm (photography of the TM sections contained more than 700 pores). The Pearson criterion value was χ^2^ = 16.98 at *f* = 9, where *f* is the number of degrees of freedom and $\;\chi_{table}^{2}=16.9190$ at the commonly used significance level of 0.05. Thus, with a validity of 0.05, the Poisson law hypothesis regarding the pore distribution over the surface of the TM could be rejected.

In total, we compiled statistics on 250 TM photos with different pore densities; TM surface parts were randomly selected from different areas. On some TM specimens, the pore distribution function was Poisson, but for most, there was an apparent deviation from the Poisson distribution (with a reliability of 0.05).

For the TM described in Table 1, the distribution function was obtained using the second method, i.e., the average value of the smallest distance between the pore centers in units *d* was calculated with the formula ${\overset{\text{¯}}{r}}_{min}=\frac{\sum r_{min}}{N\cdot d}$ (single communication method or the nearest neighbor method [32]), where *N* is the number of pores in the photo. The average distance in units *d* to the nearest pore, expected by the Poisson (random) pore distribution (Table 3), was determined with the formula ${\overset{\text{¯}}{r}}_{min}=\frac{1}{2\sqrt{n}\cdot d\;}$.

We tested the hypothesis that the nearest distance pore distribution in the TM corresponded to the probability density function [33]

(7) $$f\left(r\right)=2\pi nre^{-\pi nr^{2}}$$

The algorithm considered the edge effect: distances from pores to edges of less than five mean distances were not included in the calculations.

**Figure 6:**
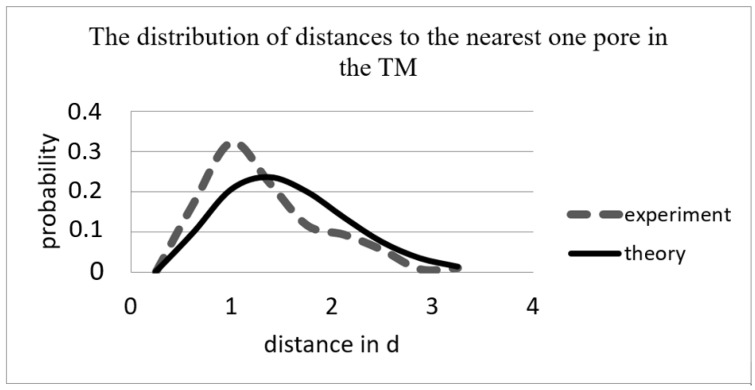
Theoretical (Formula (2)) and experimental distance distribution functions for TM with *n* = 10^6^ cm^−2^ and *d* = 4 μm (Table 2).

For the TM with a nominal radiation density of 10^6^ cm^−2^ and *d* = 4 μm, the Pearson criterion value was *χ*^2^ = 18.8 at *f* = 7, and $\;\chi_{table}^{2}=14.0671$ at a significance level of 0.05. $\;\chi^{2}<\chi_{table}^{2}$, so, for these TMs, one must reject the hypothesis of the pore distribution at the nearest distances of Formula (2) (Figure 6).

The evident difference of the curves for small distances was probably produced by the remanent stress in the biaxially oriented PET film during etching, which constricted pores toward each other. The elevated temperature of the etching was near the PET vitrification temperature.

The distribution of the smallest distances to the nearest pore did not allow for the identification of rare parts on the surface of the TM, even if the basic pore was at the border of this section. Therefore, we also calculated the pore distribution over the surface of the TM, considering the distances of up to four or six nearest pores (Table 3).

**Figure 7:**
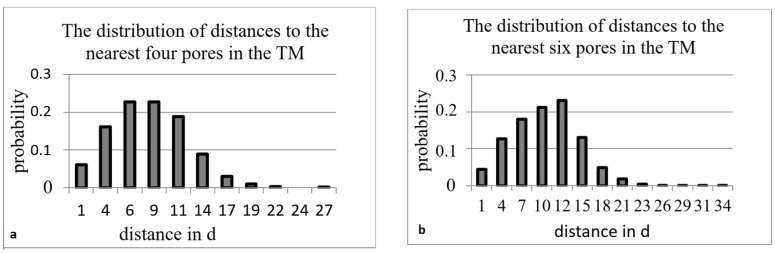
Experimental distance distribution functions for TM with *n* = 4 × 10^6^ cm^−2^ and *d* = 0.54 μm.

Examples of such distributions are given in Figure 7.

The third method of the analysis of the pore distribution determined the fractal dimension (FD) of the distribution via the net method and wavelet analysis. The programs were written in MATLAB and debugged on fractal structures with known FDs (Sierpiński triangle, Sierpiński square carpet, and ODA (limited diffusion aggregation)-cluster).

The FD of the real TM was compared with the FD of computer models of TMs at Poisson and regular pore distributions at the same density as the real TM. The results are presented in Table 6.

The FD values of objects in a plane are known to lie in a range from 1 to 2. The higher the porosity, the more FD. In our case, FD values regularity increased with increasing pore diameters at a constant pore density. At the same time, the FD values of the real TM and the computer models of the Poisson and regular distributions were practically identical. The critical distance between the pores of five diameters corresponded to FD ≈ 1.3. Thus, the FD of the pore distribution allowed us to estimate the pores’ interaction degree but was not sensitive to the type of distribution function.

**Figure 8:**
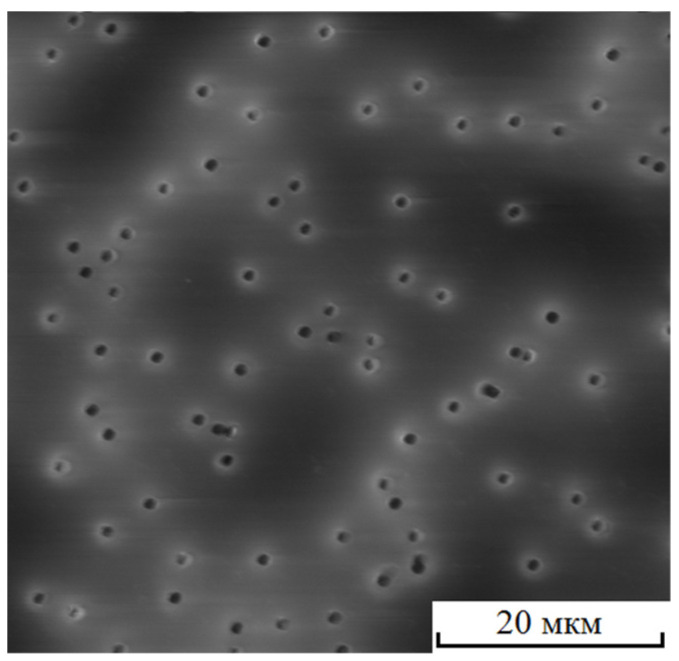
SEM image of TM with *n* = 4 × 10^6^ cm^−2^ and *d* = 1.16 µm. The frame width is 52 µm.

The visual interaction of the fields of mechanical stresses for a real membrane, such as that shown in Figure 8, was difficult to determine due to small pore sizes. We can describe the process using simulations.

For the TM shown in Figure 8, the calculated FD was about 1.4 and the average minimum distance to one nearest pore computed from SEM images was ~2.5*d*. The image clearly shows the agglomeration of pores in the form of chains.

**Figure 9:**
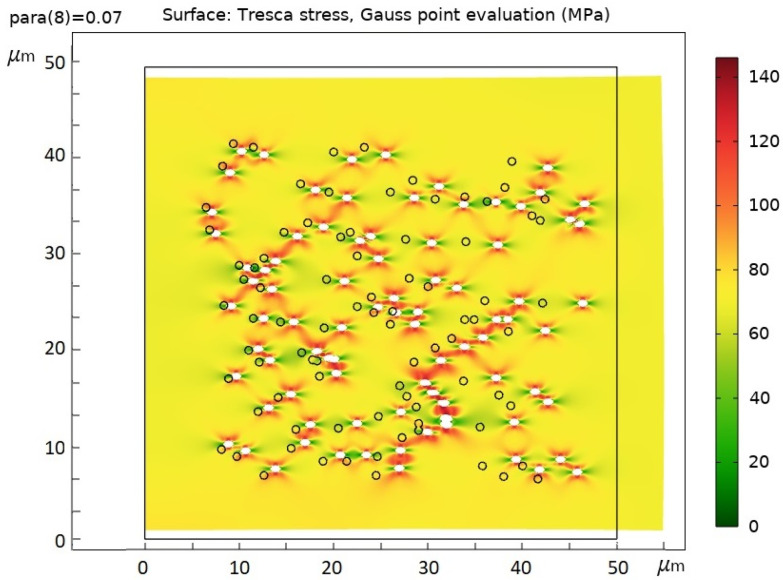
Simulation of the plastic–elastic deformation of TMs by the net-point method at one axis extension. The direction of the extension is horizontal. Dark-red zones correspond to zones with maximum stress concentrations; green represents zones of unloading.

The distribution of stress fields was obtained with finite element method in COMSOL Multiphysics 6.0 [34] for this part of the TM (Figure 9).

In Figure 9, the black circles and the frame indicate the initial positions of the pores and sample boundaries. The applied stress was 70 MPa. The maximum stress value of 140 MPa corresponds to the black–red color in the figure. Therefore, the highest stress concentration was *β* = 140/70 = 2. Dark areas in the form of series chains between the pores indicate areas of overstress in the sample and correspond to possible fracture paths. A distortion in the pores can be seen.

**Figure 10:**
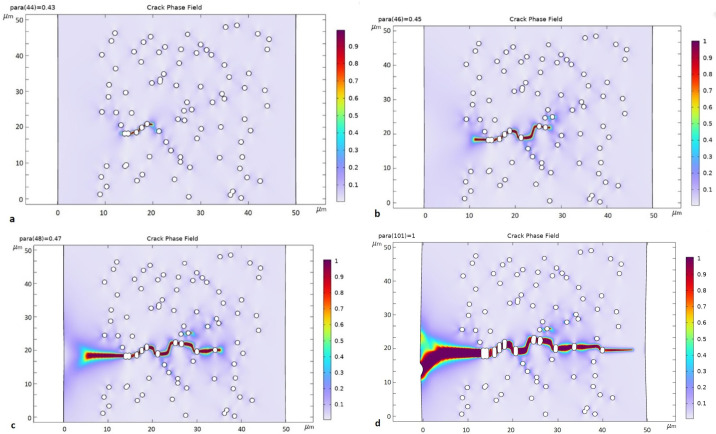
Development of crack under one-axis elongation, where the direction of deformation is vertical: (**a**) ɛ = 1.7%; (**b**) ɛ = 1.8%; (**c**) ɛ = 1.9%; (**d**) ɛ = 4%. Values of *φ* from ~0.9 to 1 (color scale) characterize a destroyed material.

The most probable crack trajectory was constructed using the phase field method in COMSOL Multiphysics 6.0 for the same part of the TM (Figure 10). In this method, the phase field is described by the field variable *φ*, which characterizes the change in the stiffness of a material; *φ* = 0 characterizes an undivided material and *φ* = 1 characterizes a destroyed material (in the approximation of brittle, i.e., actually rapid fracture) [35].

The cracking occurred on an agglomeration of pores forming a chain of closely spaced holes (Figure 10a). The further development of the crack was characterized by its rapid growth.

**Figure 11:**
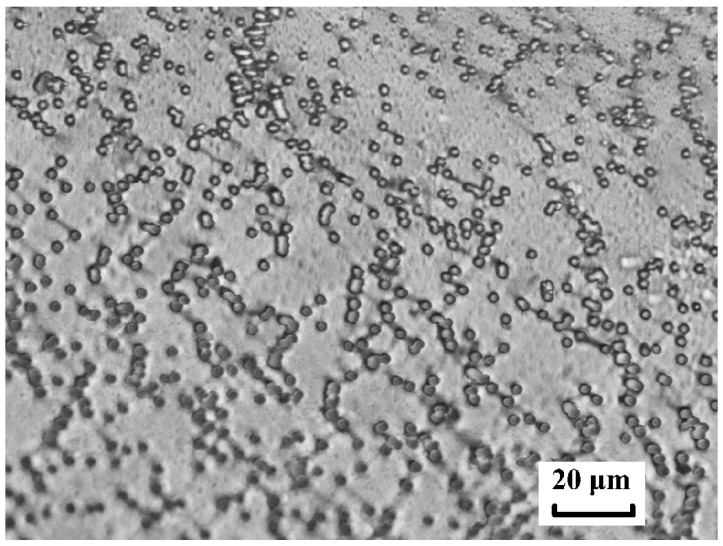
TM with *d* = 4 μm and *n* = 10^6^ cm^−2^ in polarized light, with deformation of 10%, followed by relaxation. Microcracks are visible along the pore chains. Photo was captured with a Nikon LV100 microscope.

We can also observe the microcracks’ origin in chains of closely located pores in photographs of the TM (Figure 11). However, it was only possible to catch the moment when the macrocrack appeared in a computer experiment.

## 4. Conclusions

The reduction in the working section at the tension of track-etched membranes owing to pores can be described by a formula similar to the Smith–Nielsen one. Pores are initial defects of membranes, and their coefficient of mechanical stress concentration is reduced by the evolution of the pore shape during extension. The most important factor for the formation of a system of microcracks and the following formation of a main crack during the fracture of track-etched membranes is the interaction of elastic stress fields on the pores. The interaction of mechanical stress fields is determined by the pore distribution over the surface of the membrane, which is not always subject to normal laws. The fractal dimension of the pore distribution enable the estimation of the degree of such an interaction. Relevant results can be applied to other porous materials to some extent.

## Figures and Tables

**Table 1:** Dependence of d on etching time for TM with *n* = 4 × 10^6^ cm^−2^.

Sample №	Etching Time, Min	Pore Diameter, μm	Film Thickness, μm
1	30	0.22	11.4 ± 1.1
2	50	0.54	11.0 ± 1.1
3	70	0.84	10.7 ± 1.1
4	90	1.16	10.4 ± 1.0
5	120	1.60	10.0 ± 0.9
6 *	0	-	12.0 ± 1.1

* For comparison, the experiment included test sample 6 with zero porosity (unirradiated film).

**Table 2:** Characteristics of TMs used for relative analysis.

Radiation Density, *n*, cm^−2^	Pore Diameter, μm	Film Thickness, μm
1.18 × 10^7^	0.067	11.7 ± 0.2
1.18 × 10^7^	0.105	11.7 ± 0.1
1.18 × 10^7^	0.302	11.6 ± 0.2
1.18 × 10^7^	0.487	11.4 ± 0.2
1.18 × 10^7^	0.674	11.2 ± 0.2
1.18 × 10^7^	1.221	10.8 ± 0.2
4.41 × 10^7^	0.060	11.9 ± 0.2
4.41 × 10^7^	0.158	11.9 ± 0.2
4.41 × 10^7^	0.301	11.8 ± 0.2
4.41 × 10^7^	0.772	11.2 ± 0.1
4.41 × 10^7^	0.951	11.0 ± 0.2
10^6^	4.0	10 ± 0.9

**Table 3:** Tensile strength and mechanical characteristics of tested TM.

Sample №	*d*, μm	Tensile Strength with Consideration of Reduced Working Section, *σ*, MPa	Conditional Yield Point with Consideration of Reduced Working Section, *σ*_T_, MPa	Breaking Deformation ε, %	β
1	0.22	138 ± 4	106 ± 3	19 ± 4	1.44
2	0.54	139 ± 11	111 ± 8	13 ± 1	1.43
3	0.84	140 ± 7	113 ± 6	11 ± 2	1.42
4	1.16	142 ± 5	114 ± 4	9 ± 2	1.40
5	1.60	125 ± 11	102 ± 7	5 ± 0.7	1.60
6	0	199 ± 9	106 ± 11	42 ± 2	1

**Table 4:** Relative (expressed in diameters) mean distances between pore centers for TMs with *n* = 4 × 10^6^ cm^−2^ and different diameters.

Sample №	*d*, μm	$\overset{\text{¯}}{r}\;=\;\frac{1}{\sqrt{n}\cdot d}$ (For Regular Network)	${\overset{\text{¯}}{r}}_{min}\;=\;\frac{1}{2\sqrt{n}\cdot d}$ (For Poisson Distribution)	${\overset{\text{¯}}{r}}_{min}\;=\;\frac{\sum r_{min}}{N\cdot d}$ (Calc. by SEM Images up to One Nearest Pore)	${\overset{\text{¯}}{r}}_{min}\;=\;\frac{\sum r_{min}}{N\cdot d}$ (Calc. by SEM Images up to 4 Nearest Pores)	${\overset{\text{¯}}{r}}_{min}\;=\;\frac{\sum r_{min}}{N\cdot d}$ (Calc. by SEM Images up to 6 Nearest Pores)
1	0.22	23	11.5 ± 3.4	16.9 ± 9.7	19.8 ± 9.2	23.9 ± 10.8
2	0.54	9	4.5 ± 2.0	6.9 ± 3.9	8.1 ± 3.7	9.7 ± 4.4
3	0.84	6	3 ± 1.7	4.5 ± 2.5	5.2 ± 2.4	6.2 ± 2.8
4	1.16	4	2 ± 1.4	3.2 ± 1.8	3.8 ± 1.7	4.5 ± 2.0
5	1.60	3	1.5 ± 1.2	2.3 ± 1.3	2.7 ± 1.2	3.3 ± 1.5

**Table 5:** Strength and effective stress coefficient for model samples with one and two holes.

Number of Holes	The Angle between Holes Relative to the Direction of Tension	Strength, *σ*, MPa	*β*
0	-	190 ± 9	1
1	-	161 ± 8	1.2
2	0°	128 ± 9	1.5
2	45°	112 ± 6	1.7
2	90°	118 ± 5	1.6

**Table 6:** Fractal dimension for TM surface pore distribution and TM computer models (regular network and Poisson distribution) at *n* = 4·10^6^ cm^−2^.

*d*, μm	$r_{\mathrm{Average}\;\mathrm{smallest}}=\frac{\sum r_{\mathrm{least}}}{N\cdot d}$ (calc. by SEM Images up to One Nearest Pore)	Fractal Dimension for TM	Fractal Dimension for a Model with Pores Distributed by Poisson’s Law	Fractal Dimension for a Model with Pores Distributed by Poisson’s Law
0.22	16.9 ± 9.7	1.10	1–1.10	1.10
0.54	6.9 ± 3.9	1.25	1.30	1.27
0.84	4.5 ± 2.5	1.30	1.35	1.30
1.16	3.2 ± 1.8	1.40	1.40	1.34
1.60	2.3 ± 1.3	1.55	1.50	1.50

## Data Availability

Not applicable.
